# Triglyceride deposit cardiomyovasculopathy: a rare cardiovascular disorder

**DOI:** 10.1186/s13023-019-1087-4

**Published:** 2019-06-11

**Authors:** Ming Li, Ken-ichi Hirano, Yoshihiko Ikeda, Masahiro Higashi, Chikako Hashimoto, Bo Zhang, Junji Kozawa, Koichiro Sugimura, Hideyuki Miyauchi, Akira Suzuki, Yasuhiro Hara, Atsuko Takagi, Yasuyuki Ikeda, Kazuhiro Kobayashi, Yoshiaki Futsukaichi, Nobuhiro Zaima, Satoshi Yamaguchi, Rojeet Shrestha, Hiroshi Nakamura, Katsuhiro Kawaguchi, Eiryu Sai, Shu-Ping Hui, Yusuke Nakano, Akinori Sawamura, Tohru Inaba, Yasuhiko Sakata, Yoko Yasui, Yasuyuki Nagasawa, Shintaro Kinugawa, Kazunori Shimada, Sohsuke Yamada, Hiroyuki Hao, Daisaku Nakatani, Tomomi Ide, Tetsuya Amano, Hiroaki Naito, Hironori Nagasaka, Kunihisa Kobayashi, on behalf of the Japan TGCV study group

**Affiliations:** 1 Laboratory of Cardiovascular Disease, Novel, Non-invasive, and Nutritional Therapeutics and Triglyceride Research Center (TGRC), Graduate School of Medicine, Osaka University, 6–2-4, Furuedai, Suita, Osaka, 565-0874 Japan; 2 Department of Pathology, National Cerebral and Cardiovascular Center, 5–7-1, Fujishirodai, Suita, Osaka, 565-8565 Japan; 3 Department of Radiology, National Hospital Organization Osaka National Hospital, 2–1-14, Hoenzaka, Chuo-ku, Osaka, 540-0006 Japan; 4 Department of Biochemistry, Fukuoka University Medical School, 7–45–1, Nanakuma, Jonan-ku, Fukuoka, 814-0180 Japan; 5 Department of Metabolic Medicine, Graduate School of Medicine, Osaka University, 2–2, Yamadaoka, Suita, Osaka, 565-0871 Japan; 6 Department of Cardiovascular Medicine, Tohoku University Graduate School of Medicine, 1–1, Seiryomachi, Aoba-ku, Sendai, Miyagi 980-8574 Japan; 7 Department of Cardiovascular Medicine, Chiba University Graduate School of Medicine, 1–8-1, Inohara, Chuo-ku, Chiba, 260-8670 Japan; 8 Division of Molecular Brain Science, Kobe University Graduate School of Medicine, 7–5-1, Kusunoki-cho, Chuo-ku, Kobe, 650-0017 Japan; 9 Department of Applied Biological Chemistry, Graduate School of Agriculture, Kindai University, 3327–204, Nakamachi, Nara, 631-8505 Japan; 10 Faculty of Health Sciences, Hokkaido University, Kita-12, Nishi-5, Sapporo, 060-0812 Japan; 11 Kure Medical Center and Chugoku Cancer Center, National Hospital Organization, 3–1, Aoyama-cho, Kure, Hiroshima, 737-0023 Japan; 12 Department of Cardiovascular Medicine, Komaki City Hospital, 1–20, Jobushi, Komaki, Aichi 485-8520 Japan; 13 Department of Cardiovascular Medicine, Juntendo University Graduate School of Medicine, 2–1-1, Hongo, Bunkyo-ku, Tokyo, 113-8421 Japan; 14 Department of Cardiology, Aichi Medical University, 1–1 Yazakokarimata, Nagakute, Aichi 480-1195 Japan; 15 Department of Cardiovascular Medicine, Graduate School of Medicine, Nagoya University, 65 Tsurumai, Showa-ku, Nagoya, Aichi 466-8560 Japan; 16 Department of Infection Control and Laboratory Medicine, Kyoto Prefectural University of Medicine, 465, Kajii-cho, Kawaramachi-Hirokoji, Kamigyo-ku, Kyoto, 602-8566 Japan; 17 Faculty of Human Life Science, Osaka City University, 3–3-138, Sugimoto, Sumiyoshi-ku, Osaka, 558-8585 Japan; 18 Department of Internal Medicine, Division of Kidney and Dialysis, Hyogo College of Medicine, 1–1 Mukogawa-cho, Nishinomiya, Hyogo 663-8501 Japan; 19 Department of Cardiovascular Medicine, Hokkaido University Graduate School of Medicine, Kita-15, Nishi-7, Kita-ku, Sapporo, 060-8638 Japan; 20 Department of Pathology and Laboratory Medicine, Kanazawa Medical University, 1–1 Daigaku, Uchinada, Ishikawa 920-0293 Japan; 21 Department of Pathology, Nihon University School of Medicine, 30–1 Ohyaguchikami-cho, Itabashi-ku, Tokyo, 173-8610 Japan; 22 Center for Global Health, Department of Medical Innovation, Osaka University Hospital.4F Center of Medical Innovation and Translational Research, 2–2 Yamadaoka, Suita, Osaka, 565-0871 Japan; 23 Department of Cardiovascular Medicine, Osaka University Graduate School of Medicine, 2–2 (A8) Yamadaoka Suita, Osaka, 565-0871 Japan; 24 Department of Cardiovascular Medicine, Graduate School of Medicine, Kyushu University, 3–1–1, Maidashi, Higashi-ku, Fukuoka, 812-8582 Japan; 25 Department of Radiology, Nippon Life Hospital, 2–1-54, Enokojima, Nishi-ku, Osaka, 550-0006 Japan; 26 Department of Pediatrics, Takarazuka City Hospital, 4–5-1, Obama, Takarazuka, Hyogo 665-0827 Japan; 27 Department of Endocrinology and Diabetes Mellitus, Fukuoka University Chikushi Hospital, 1–1-1, Zokumyoin, Chikushino, Fukuoka, 818-8502 Japan

**Keywords:** Adipose triglyceride lipase, Atherosclerosis, Rare disease, Triglyceride-deposit cardiomyovasculopathy, Triglyceride metabolism

## Abstract

Triglyceride deposit cardiomyovasculopathy (TGCV) is a phenotype primarily reported in patients carrying genetic mutations in *PNPLA2* encoding adipose triglyceride lipase (ATGL) which releases long chain fatty acid (LCFA) as a major energy source by the intracellular TG hydrolysis. These patients suffered from intractable heart failure requiring cardiac transplantation. Moreover, we identified TGCV patients without *PNPLA2* mutations based on pathological and clinical studies*.* We provided the diagnostic criteria, in which TGCV with and without *PNPLA2* mutations were designated as primary TGCV (P-TGCV) and idiopathic TGCV (I-TGCV), respectively. We hereby report clinical profiles of TGCV patients. Between 2014 and 2018, 7 P-TGCV and 18 I-TGCV Japanese patients have been registered in the International Registry. Patients with I-TGCV, of which etiologies and causes are not known yet, suffered from adult-onset severe heart disease, including heart failure and coronary artery disease, associated with a marked reduction in ATGL activity and myocardial washout rate of LCFA tracer, as similar to those with P-TGCV. The present first registry-based study showed that TGCV is an intractable, at least at the moment, and heterogeneous cardiovascular disorder.

## Triglyceride (TG) and orphan diseases

TG is a major energy source for mammals. In normal condition, TG is either received via the diet, or synthesized endogenously and stored in adipose tissues. When required, TG is hydrolyzed by various enzymes called lipases and releases long-chain fatty acid (LCFA), which is delivered to non-adipose tissues for the production of ATP. It has been known that the ectopic TG deposition in non-adipose tissues causes some orphan diseases. In 1953, Jordans reported two brothers with phenotype of skeletal myopathy and vacuolar formation of peripheral leukocytes, called Jordans’ anomaly [1]. Fifty years later, Fischer et al. found that this phenotype is associated with mutations in *PNPLA2* [2] encoding adipose TG lipase (ATGL) [3, 4], an essential molecule located in cytoplasmic lipid droplets for the intracellular TG hydrolysis [5, 6], and designated this phenotype as neutral lipid storage disease with myopathy (NLSD-M). Clinical manifestations of NLSD-M appeared variable from mild to severe symptoms [7–13], which could be at least partially explained by function of mutated ATGL proteins [14]. Another phenotype of NLSD involving the skin was reported as NLSD with ichthyosis (NLSD-I) by Chanarin and Dorfman in the 1970s [15–17]. The genetic cause of NLSD-I was found to be mutations in *ABHD5* encoding CGI-58, a co-enzyme of ATGL [18]. Using skin fibroblasts and iPS cells from patients with NLSDs, unique intracellular metabolism of TG has been extensively analyzed. These cell-biological experiments showed that cytoplasmic lipid droplets are dynamic cellular organelles interacting with ATGL, CGI-58, and other proteins, and could be a therapeutic target [19–23].

## Discovery of TG-deposit cardiomyovasculopathy (TGCV) with *PNPLA2* (ATGL) mutation

Since the early 1980s, patients with Jordans’ anomaly and severe heart failure (HF), though very rare, had been reported in Japan [24]. In the early 2000s, our institution started to take care of two patients with severe HF and vacuolar formation in peripheral leukocytes. HF was progressive and intractable, and a couple of years later, they became candidates for cardiac transplantation (CTx). Preoperative examination of their hearts exhibited dilated cardiomyopathy-like morphology in chest X-ray and ultrasonography; however, endomyocardial biopsy specimens showed neutral lipid deposition in cardiomyocytes [25]. When they underwent CTxs, pathological and biochemical analyses of their explanted hearts were performed, demonstrating that their coronary arteries showed unusual coronary atherosclerosis with TG deposition in endothelial and smooth muscle cells (SMCs). We named this novel phenotype as TGCV [26–28]. These patients were identified as homozygous for genetic mutations in *PNPLA2* encoding ATGL, which is also known to be responsible for NLSD-M as described above [2].

## Postmortem analyses revealed undiagnosed individuals with TGCV

**Fig. 1:**
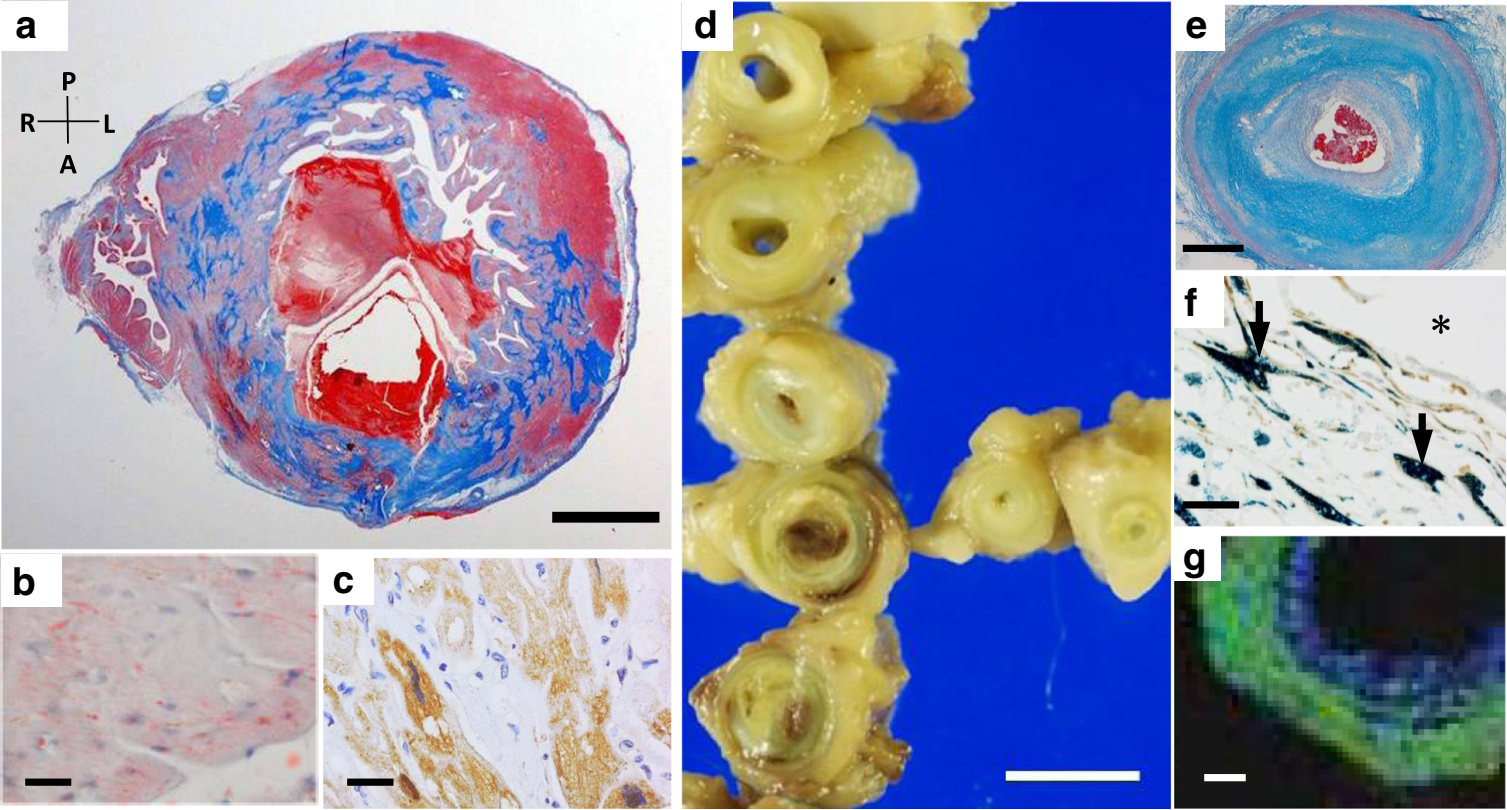
Pathological analysis of the autopsied heart of a 38-year-old man with TGCV phenotype without PNPLA2 mutation. Panel **a** The transverse section of the autopsied heart stained with Masson’s trichrome showed circumferential patchy fibrosis of the left ventricular wall. The letters A, L, R, and P denotes anterior, left, right, and posterior, respectively. Panel **b** Lipid droplets (LDs) stained with oil red O in the cytoplasm of cardiomyocytes. Panel **c** Immunostaining for ATGL (Cell Signaling, Danvers, MA). Cardiomyocytes showed positive reactivity for ATGL. Panel **d** Coronary arteries with diffuse concentric-type stenosis. Panel **e** The transverse section of coronary artery was stained with Masson’s trichrome. Coronary artery revealed intimal thickening and fibroatheromatous lesions. Panel **f** Double-staining of Sudan black B and α-smooth muscle actin (Dako, Tokyo, Japan). Smooth muscle cells (brown color) with lipid droplet (blue color) distributed diffusely in the media and intima (arrows in Panel **f**). Asterisk represents the vascular lumen. Panel **g** TG (*m/z* 879.7) was identified as green and blue colors depending on the intensity. TG signals were diffusely detected in the arterial wall by imaging mass spectrometry. Green color denotes relatively higher intensity of TG than blue color. Myocardial and coronary TG contents (3.64 and 19.44 mg/g of tissue, respectively) were higher in this patient, compared with each of control group (1.4 ± 1.0, and 6.2 ± 4.8 mg/g of tissue, respectively). The detailed clinical profile of this patient is reported as Case 10 in the reference [29]. Scale bars: 1 cm in Panel **a**, 20 μm in Panel **b** and **c**, 5 mm in Panel **d**, 1 mm in Panel **e**, 20 μm in Panel **f**, 200 μm in Panel **g**

Retrospective postmortem analyses of autopsied cases identified individuals with TGCV phenotype who had TG deposit in both myocardium and coronary arteries, as presented in Fig. 1. A 38-year-old man suddenly died irrespective of intensive treatment for coronary artery disease (CAD) and HF. His heart was heavy in weight and hypertrophied with multiple myocardial fibrous scars. Coronary arteries showed diffuse and concentric stenosis in multi-vessels. Biochemical analyses and imaging mass spectrometry showed TG deposition in both myocardium and coronary arteries [29, 30]. TG-deposit SMCs were observed in his renal and mesenteric arteries as well (data not shown). These data mimic genetic ATGL deficiency; however, the immunoreactive mass of ATGL was detected, and the genetic test using genomic DNA extracted from stored specimens showed no mutation in all exons and exon/intron boundaries of *PNPLA2* gene (data not shown). In addition, pathological records showed that he did not have skeletal myopathy.

## Development of diagnostic methods for TGCV

The above postmortem studies suggested that it is difficult to diagnose TGCV, and many undiagnosed patients should have died, which motivated us to develop diagnostic tools and methods for TGCV. We reported that myocardial scintigraphy with iodine-123-β-methyl iodophenyl-pentadecanoic acid (BMIPP) [31, 32], a radioactive analogue of LCFA, was useful in detecting abnormal LCFA metabolism in patients with TGCV [33, 34]. In addition, we reported the use of automated hematology analyzers to detect Jordans’ anomaly in patients with *PNPLA2* mutation [35–37]. Recently, we developed CT-based TG imaging to detect myocardial and coronary TG deposition [34, 38] and selective immunoinactivation assay to measure functional ATGL activities using peripheral leukocytes [39].

## Nomenclature, definition, and classification of TGCV

**Fig. 2:**
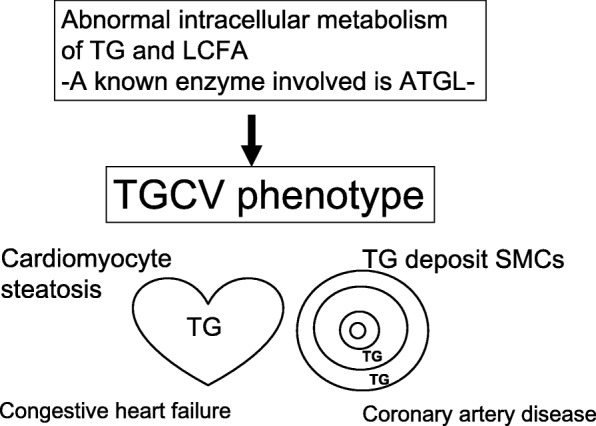
Schematic presentation of the disease concept for TGCV

It is well known that disease nomenclature is made not only by their genotypes, but also by their phenotypes in many diseases and by discoverer’s names in some diseases. The nomenclature of TGCV was made by its phenotype that TG accumulated in both myocardium and coronary arteries, resulting from abnormal intracellular metabolism of TG and LCFA (Fig. 2) [26–28]. ATGL is a known enzyme involved in the phenotypic expression of TGCV. The Japan TGCV study group provided the diagnostic criteria for TGCV, in which TGCV with and without *PNPLA2* mutations was designated as primary TGCV (P-TGCV) and idiopathic TGCV (I-TGCV), respectively [40–42].

## Pathophysiology of TGCV

**Fig. 3:**
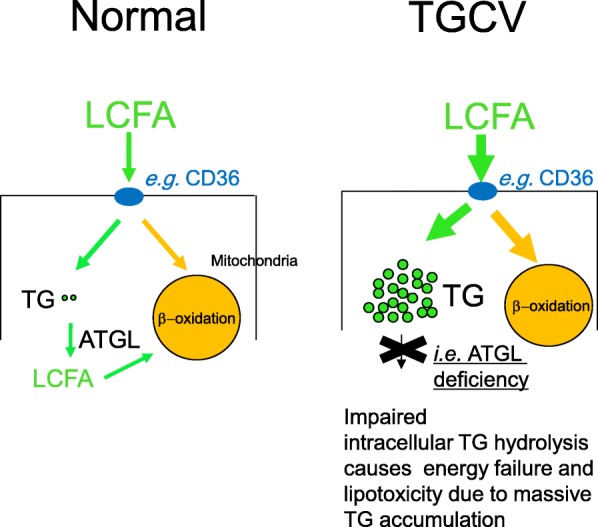
A pathophysiological model for TGCV. Genetic and acquired ATGL deficiency and other causes result in abnormal intracellular metabolism of TG and LCFA, leading to cardiomyocyte steatosis and TG-deposit SMCs. In normal condition (left panel), LCFA is taken up through LCFA transporters and receptors such as CD36 and some of them are transported to mitochondria for β-oxidation and the remaining LCFAs are utilized as a source for TG and rapidly hydrolyzed by intracellular lipases such as ATGL. In TGCV (right panel), LCFAs are taken up and used for the synthesis of TG which can not be hydrolyzed, leading to massive TG accumulation

**Fig. 4:**
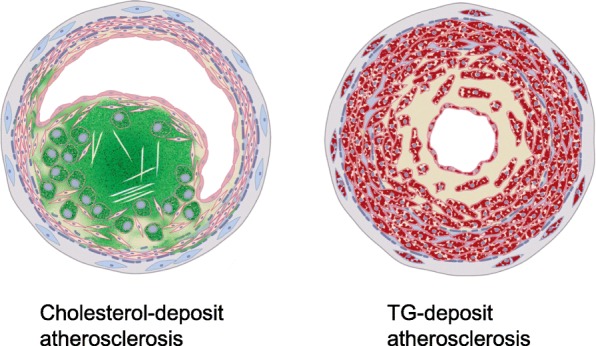
Schemes for cholesterol- (Left) and TG-deposit atherosclerosis (Right). In cholesterol-deposit atherosclerosis, cholesterol (green) accumulates in macrophages, leading to eccentric stenosis. In TG-deposit atherosclerosis, TG (red) accumulates in SMCs, leading to concentric stenosis, which is a major feature of TGCV

The pathophysiological schema of TGCV is shown in Fig. 3. In normal condition (left panel, Fig. 3), LCFAs are taken up through transporters and receptors such as CD36. Some are transported to the mitochondria for β-oxidation, and the remaining LCFAs are utilized as a source of TG and rapidly hydrolyzed by intracellular lipases such as ATGL. In TGCV (right panel, Fig. 3), LCFAs are taken up and used to synthesize TG that cannot be hydrolyzed due to ATGL insufficiency, leading to energy failure and lipotoxicity with massive TG accumulation [28, 43]. It is emphasized that TG-deposit atherosclerosis is an important characteristic of TGCV [44] and distinct from usual cholesterol-deposit atherosclerosis, because the former showed diffuse and concentric narrowing formed by TG-deposit SMCs, whereas the latter showed discrete and eccentric stenosis initiated by the response to injury in the endothelium and accumulation of cholesterol-laden macrophages [45] (Fig. 4). We reported that TG-deposit SMCs and endothelial cells had pro-inflammatory and vulnerable phenotype in vitro [46, 47].

## A clinical case presentation of I-TGCV

**Fig. 5:**
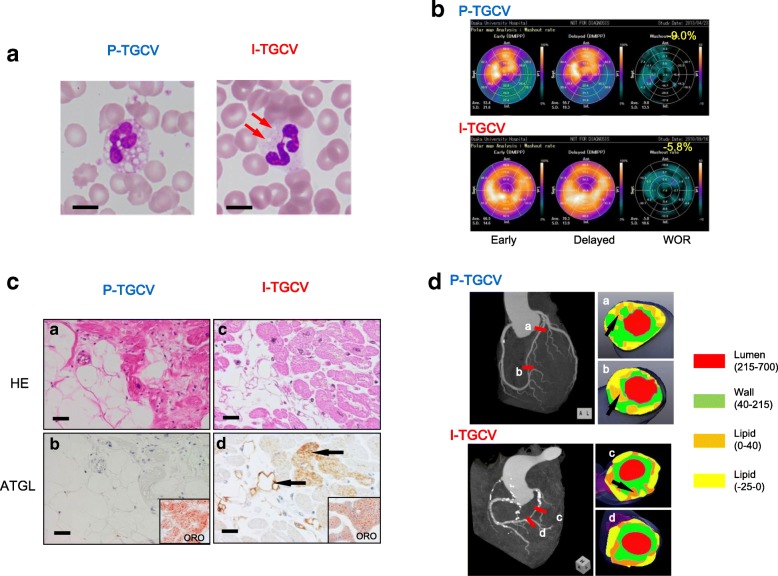
Laboratory and imaging examinations for TGCV. **a** Representative images of May-Giemsa staining of blood smears were shown from patients with P-TGCV and I-TGCV. **b** Bull’s eye images for BMIPP scintigrams from patients with P-TGCV and I-TGCV. The first scan was performed 20 min post-injection to determine early BMIPP uptake, and the second scan was performed 200 min later to study delayed uptake using myocardial SPECT after patients were injected with ^123^I-BMIPP. WOR was calculated with the Hear Risk View-S (HRSV) software as the difference between early and delayed images (reference value, 19.4 ± 3.2%). **c** A patient with P-TGCV showed numerous vacuoles (Panel a, H&E) in cardiomyocytes that stained positively for oil red O (ORO) (inset in Panel b). Furthermore, no positive reactivity for ATGL observed in any of cell types (Panel b, ATGL). Cardiomyocytes of patients with I-TGCV showed numerous vacuoles (Panel c, HE) filled with stained lipid (inset in Panel d, ORO), whereas positive reactivity for ATGL observed not only in adipocytes but also in cardiomyocytes (arrows in Panel d, ATGL). Scale bars: Panels a-d, 30 μm. **d** Coronary CT angiograms (CTA) from patients with P-TGCV and I-TGCV are shown. Bars in CTA correspond to Panels a-d, which are short axial sections of the left anterior descending coronary artery. The segmentation of the coronary artery lumen and wall was done using a workstation (Ziostation 2, Ziosoft, Japan). Constitutive components were classified into 4 colors with the original analysis software as follows. Colors indicate the CT number (yellow, − 25–0; orange, 0–40; green, 40–215; red, 215–700 Hounsfield unit [HU] (M@XNET, Tokyo, Japan) in Panels a-d. Yellow or orange areas indicate lipid components, red shows blood, and green shows the arterial wall without calcification or lipids. Black arrows in Panels a, b, and c indicate outside-in protrusion, which is the characteristics for TGCV

A 58-year-old woman was referred to our hospital due to sudden chest tightness with ST-segment elevation in the electrocardiogram, followed by cardiopulmonary arrest. Under the diagnosis of acute myocardial infarction, she underwent coronary artery bypass grafting (CABG). Past history included type 2 diabetes mellitus requiring insulin treatment and hemodialysis. Cytoplasmic vacuoles in her peripheral polymorphonuclear leukocytes were observed less frequently (< 10% of neutrophils), compared with that in genetic ATGL deficiency (panel A in Fig. 5). ATGL activity in peripheral leukocytes was very low, comparable to that of genetic ATGL deficiency, as shown in Table 1. Myocardial washout rate (WOR) of BMIPP was defective in scintigraphy (panel B in Fig. 5). Pathological analyses of endomyocardial biopsy specimens demonstrated numerous vacuoles filled with stained lipid but positive reactivity for ATGL in cardiomyocytes and adipocytes (right, panel C in Fig. 5). Coronary CT angiogram showed diffuse narrowing coronary arteries, and in TG imaging [25], outside-in involvement of diffuse and abundant lipid components expressed as low CT numbers was seen within the wall in a peninsular pattern (arrows in panel D in Fig. 5). Her laboratory data and imaging tests were similar to those observed in TGCV with genetic ATGL deficiency, except for the conserved expression of ATGL protein in the myocardium. However, it is noted that the case was clinically distinct from genetic ATGL deficiency because there was no skeletal myopathy and no elevation of MM type creatine kinase. Genetic tests showed no mutations or substitutions in any of the exons or intron/exon boundaries of genes encoding ATGL, 1-acylglycerol-3-phosphate O-acyltransferase, hormone-sensitive lipase, or GOS2 (data not shown).

**Table 1:** Patients’ characteristics of Primary and Idiopathic TGCV

	Primary	Idiopathic
	*n* = 7	*n* = 18
General Status		
Age (years)	55.7 ± 12.7	64.6 ± 14.7
Sex (female, male) (n)	(2, 5)	(9, 9)
BMI (kg/m2)	19.4 ± 3.4	25.4 ± 5.0
Family history for CVD	7	13
ATGL expression		
PNPLA2 mutation	Yes	No**
ATGL activities in leukocytes (nmol/h/mg)*	5.3 ± 8.3	12 ± 9
(reference value 52 ± 13 nmol/h/mg)		
Vacuole formation in polymorphonuclear leukocytes (%)	~ 100%	< 10%
Heart disease		
Mean age of symptom onset (years)	37.7 ± 9.2	55.9 ± 12.5
Angina at rest (n)	3	10
Dyspnea or palpitation (n)	4	8
Clinical diagnosis at registration		
Angina pectoris	1	13
(rest, effort) (n)	(1,0)	(11, 2)
Heart failure (n)	5	8
Critical arrhythmia (n)	4	1
History of myocardial infarction (n)	0	4
NYHA classification (I, II, III, IV) (n)	(1, 1, 2, 3)	(2, 5, 11, 0)
Coronary angiography or CT angiogram		
Affected branch (single vessel, multivessels) (n)	(0, 5)	(4,14)
Diffuse narrowing (n)	5	18
Washout rate in BMIPP scintigram (%)	一3.2 ± 4.8	1.4 ± 8
(reference value 19.4 ± 3.2%)		
Treatment history		
Percutaneous coronary intervention (n)	0	7
Coronary artery bypass grafting (n)	0	5
Cardiac transplantation (n)	2	0
Comorbidity		
Skin lesions (n)	0	0
Skeletal myopathy (n)	7	0
Diabetes mellitus (n)	2	15
Outcome		
Death (n)	5	3
(before, after registration)	(3, 2)	(2, 1)

*Three patients with P-TGCV and fourteen with I-TGCV were enrolled

We did not have opportunity for the measurement in the remaing four patients with P-TGCV and four with I-TGCV

**Two patients were dismissed before the genetic analysis

The Japan TGCV study group certified I-TGCV according to the diagnostic guideline

Abbreviations: *CT* Computed tomography, *CVD* Cardiovascular disease, *TGCV* Triglyceride deposit cardiomyovasculopathy

## Clinical characteristics of P- and I-TGCV

Table 1 shows the clinical characteristics of 7 and 18 patients with P- and I-TGCV, respectively, registered to the international registry for NLSD and TGCV between February 2014 and March 2018 in Japan. Both TGCV types were adult onset with chest pain at rest or dyspnea and palpitation. Most patients with either types of TGCV developed severe HF or CAD with diffuse narrowing multivessel lesions or both. Myocardial metabolism of LCFA, detected by WOR of BMIPP and ATGL activities in peripheral leukocytes, was reduced in both TGCV types. Most patients with P-TGCV developed intractable and critical HF, as reported recently [26, 48, 49]. Two of them underwent CTx [26, 48]. Many patients with I-TGCV required percutaneous coronary intervention and CABG. As comorbidity, neither type of TGCV had skin lesions, which suggests that TGCV is not associated with NLSD-I. All patients with P-TGCV had skeletal myopathy, whereas none of those with I-TGCV did. Five of 7 and 3 of 18 registered patients with P- and I-TGCV, respectively, died.

## Differential diagnosis of TGCV

Myocardial disorders such as dilated cardiomyopathy, hypertrophic cardiomyopathy, arrhythmogenic right ventricular cardiomyopathy, mitochondrial cardiomyopathy, alcoholic heart disease, and metabolic myocardial disorders (e.g., Fabry disease, Pompe disease, cholesteryl ester storage disease) need to be differentiated from TGCV [41, 42].

Furthermore, known diabetic and metabolic heart diseases need to be differentiated from TGCV. One is diabetic cardiomyopathy, which was originally defined as cardiomyopathy without significant stenosis in epicardial coronary arteries [50]. Another concept is epicardial fat accumulation, which is the oeverdeposition of TG in physiological tissues. TGCV is distinct from these two entities because TGCV is characterized by the ectopic deposition of TG in the cardiomyocytes and SMCs with apparent involvement of epicardial coronary arteries, as shown in Figs. 1 and 5.

## Academia-initiated development of specific treatment for TGCV

We found that the chow with tricaprin, TG form of capric acid, improved LCFA metabolism, lipid deposition, cardiac function, and life span in ATGL-targeted mice [4], raising a therapeutic hypothesis that capric acid may be an alternative energy source and reduce TG deposition and lipotoxicity in TGCV [51]. Based upon these data, the Osaka University Hospital manufactured GMP-graded capsules containing the active gradients called CNT-01. We developed the assay to measure plasma capric acid levels [52, 53]. After finishing toxicity tests using rats and dogs required, we are finally conducting investigator-initiated clinical trials.

## Comparison between NLSD-I, NLSD-M, and TGCV

**Fig. 6:**
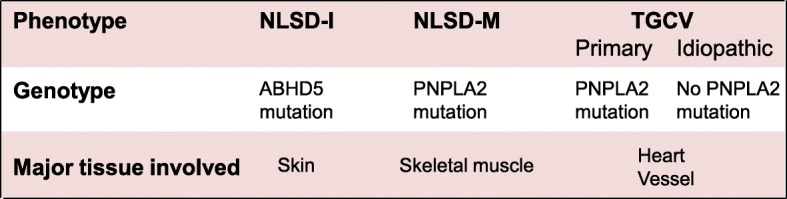
Relationship between TGCV and NLSDs. Comparison of phenotype and genotype between NLSD-I, NLSD-M and TGCV

As mentioned above, the nomenclature of TGCV was made by its phenotype that TG accumulated in both myocardium and coronary arteries, resulting from abnormal intracellular metabolism of TG and LCFA (Figs. 2 and 3). As described in the first paragraph in this letter, there have been known related disorders; NLSD-M and NLSD-I. Figure 6 shows the comparison of phenotype and genotype between TGCV and NLSDs. NLSD-M and NLSD-I are caused by mutations in *PNPLA2* and *ABHD5*, mainly involved in the skeletal muscle and skin, respectively. Genotype of P-TGCV is known to be *PNPLA2* mutation which is responsible for NLSD-M as well.

## Issues to be resolved

The following points are important focus for future researches:

1. Possible clinical continuum between P-TGCV and NLSD-M
  As mentioned above, both P-TGCV and NLSD-M is caused by genetic ATGL deficiency. It would be of interest to know whether patients with NLSD-M have TG-deposit atherosclerosis, which is the important feature for P-TGCV.
2. Etiologies of I-TGCV and its prevalence in countries other than Japan
  As shown in Table 1, 13 out of 18 patients with I-TGCV had family history of cardiovascular disease, suggesting that any genetic factors might be involved in the pathogenesis of I-TGCV. The mechanism underlying downregulation of ATGL activities of I-TGCV and possible involvement of other lipases and related enzymes is of significance to elucidate. In order to elucidate these issues, the development of screening methods for the diagnosis of I-TGCV is under way in our laboratory.

## Conclusions

TGCV is a severe cardiovascular disorder named by its phenotype of cardiomyovascular TG deposition, of which etiologies seem heterogeneous.

## Methods

1. Pathological, laboratory, and clinical imaging
  Standard procedures were performed as described (please see legends of Figs. 1 and 5).
2. International registry for NLSD/TGCV
  On the World Rare Disease Day 2014, we launched the international registry for neutral lipid storage diseases, TG-deposit cardiomyovasculopathy, and related disorders (Clinical Trial gov. NCT02830763).The present patients with TGCV were registered according to the study protocol after obtaining written consent. The protocol was approved by the Osaka University Hospital Ethical Committee (approval no. 13204).

## Abbreviations

ATGL: Adipose triglyceride lipase
BMIPP: Iodine-123-β-methyl iodophenyl-pentadecanoic acid
CABG: Coronary artery bypass grafting
CAD: Coronary artery disease
CTA: CT angiograms
CTx: Cardiac transplantion
HF: Heart failure
Hu: Hounsfield unit
LCFA: Long-chain fatty acid
NLSD: Neutral lipid storage disease
NLSD-I: NLSD with ichthyosis
NLSD-M: NLSD with myopathy
SMCs: Smooth muscle cells
TG: Triglyceride
TGCV: Triglyceride-deposit cardiomyovasculopathy
WOR: Washout rate
